# Distribution and Retention Trends of Obstetricians and Gynecologists in Japan: A Longitudinal Study

**DOI:** 10.31662/jmaj.2020-0125

**Published:** 2021-07-06

**Authors:** Masatoshi Ishikawa

**Affiliations:** 1 Harvard T.H. Chan School of Public Health, Harvard University, Boston, USA; 2 Research and Development Center for Health Services Tsukuba, University of Tsukuba, Tsukuba, Japan

**Keywords:** censuses, distribution, Japan, longitudinal study, obstetricians and gynecologists

## Abstract

**Introduction:**

This longitudinal study aimed to investigate the distribution and retention of obstetricians and gynecologists in Japan.

**Methods:**

I used descriptive statistics and multivariate logistic regression to analyze data from National Census surveys administered during 1996-2016.

**Results:**

Between 1996 and 2016, the number of obstetricians and gynecologists increased by 6% and urban physicians by 15%, whereas the number of rural physicians decreased by 25%. The annual retention rate, which was calculated using the square root of the biannual rates [the number of physicians still working as obstetricians and gynecologists at the time of the subsequent survey (e.g., in 1998) divided by the number of obstetricians and gynecologists in the original survey (e.g., in 1996)], was >90%. Obstetricians and gynecologists were less likely to continue to work as obstetricians and gynecologists after 30-44 years of experience (1996-2006 cohort: OR = 0.20, 95% CI = 0.17-0.25; 2006-2016 cohort: OR = 0.32, 95% CI = 0.25-0.41) and >45 years of experience (1996-2006 cohort: OR = 0.14, 95% CI = 0.11-0.17; 2006-2016 cohort: OR = 0.11, 95% CI = 0.08-0.15). The odds were lower for rural obstetricians and gynecologists (1996-2006 cohort: OR = 0.65, 95% CI = 0.51-0.82; 2006-2016 cohort: OR = 0.59, 95% CI = 0.43-0.80). As the number of female physicians increased, the number of practicing obstetricians and gynecologists also increased. In 2004, the mandatory postgraduate clinical training that was newly implemented caused a drop in the number of young doctors; however, this reversed in 2006. Rural to urban migration was steady, and the working hours were consistently long. To stabilize high retention rates, the working environments need to be improved.

**Conclusions:**

The present study clearly indicated the trend of the distribution of obstetricians and gynecologists in Japan. The result may be especially important for the health policy making in Japan.

## Introduction

The uneven distribution of physicians with certain clinical specialties is a common issue worldwide ^(1)^. With Japan’s 2018 revisions to the Medical Service Act and Medical Practitioners’ Act, all prefectures were required to formulate physician retention plans by 2019. These data are then utilized to establish measures that can rectify maldistribution ^(2)^. The shortage of obstetricians and gynecologists (OBGYNs) is especially severe compared with that of other specialties. This makes the issue critical in the Japanese healthcare policy ^(3)^.

Over the last 20 years, the number of female OBGYNs has increased in Japan ^(4)^. Compared with their male counterparts, women tend to work fewer hours and are preferentially tasked with childcare. Some argue that these factors contribute to the shortage of OBGYNs in the country ^(5)^. Moreover, OBGYNs tend to focus on urban areas, which is a problematic trend common to all clinical specialties ^(6), (7), (8)^.

Japan’s perinatal mortality rate is among the lowest in the developed world, and it has one of the best worldwide perinatal healthcare systems ^(9)^. However, it is characterized by numerous decentralized, relatively small-scale delivery units ^(10)^. There are few physicians who choose to specialize as OBGYNs, thus magnifying regional inequality between urban and rural areas. This maldistribution has led experts at the Japan Society of Obstetrics and Gynecology (JSOG) and the Japan Association of Obstetricians and Gynecologists (JAOG) to propose consolidating key OBGYN hospitals while improving the working environment ^(11)^.

Some insights into recent OBGYN trends can be developed from the Survey of Physicians, Dentists, and Pharmacists, which is administered to all physicians in Japan every 2 years by the Ministry of Health, Labour and Welfare (MHLW). However, detailed statistics and individual-level data concerning changes in OBGYN demographics and career paths have not been publicly disclosed ^(12)^.

Nevertheless, a study that obtained detailed MHLW data found that from 1972 to 2004, very few young OBGYNs were active, and their numbers hardly changed during those 30 years ^(13)^. Even so, we know little about how OBGYN regional maldistribution has changed since 2004 and about the career paths these professionals have taken in the interim.

Fortunately, we can use the Survey of Physicians, Dentists, and Pharmacists conducted by MHLW to determine which physicians choose to specialize as OBGYNs. Such research generates basic data for developing measures to address uneven OBGYN distribution, such as physician retention plans.

Therefore, this study aimed to evaluate the geographical maldistribution and retention trends of OBGYNs in Japan while identifying the factors associated with both variables. In conclusion, I describe the potential policy implications of my findings.

## Materials and Methods

With the approval from the MHLW, I used data from the 1996-2016 Surveys of Physicians, Dentists, and Pharmacists. The Medical Practitioners’ Act requires all physicians to report their status every 2 years. The response rate was estimated to be around 90% ^(14)^.

I developed two cohort datasets (1996-2006 and 2006-2016) using physician registration numbers and analyzed their geographical movement patterns. When creating the cohort dataset, I analyzed the physicians who responded to the surveys in both years. Moreover, in the original data obtained from the MHLW, there were no incomplete or missing data.

The following aspects were evaluated for each physician: registration number, sex, age, years of experience, workplace type (municipality and medical institution), and area of practice (e.g., OBGYN). In 1996 and 2016, 12 402 (5.2% of the total) and 13 154 (4.1%) physicians were OBGYNs, respectively.

Municipality borders were based on 2016 delineations to adjust for mergers. In total, 344 secondary medical areas (SMAs) were identified, which were then classified into three categories based on the 2016 population size and density: urban, intermediate, and rural. Japan does not have rurality criteria comparable to the United States Office of Management and Budget ^(15)^. Thus, this study used the classification that the MHLW employs for position statements with regard to physician demand ^(16)^. The urban group comprised places with a population of at least 1 million or a population density of at least 2000 individuals/km^2^. The intermediate group was defined as places with at least 100 000 people or a density of at least 200 individuals/km^2^. The remaining areas were categorized in the rural group. Physicians in the same SMA group throughout the study period were considered retained.

The number of physicians per 100 000 people in each SMA group was calculated using data from the National Census ^(17)^. To account for the fact that the physician and population data were taken from different years, the physician data from 1996, 2006, and 2016 were matched to population data from 1995, 2005, and 2015, respectively.

Physicians were also classified based on the institutions of employment: clinic, university hospital, non-university hospital, and others. In Japan, a clinic is a medical institution with fewer than 20 inpatient beds, whereas a hospital has 20 or more inpatient beds.

The OBGYN demographic and professional characteristics for these 3 years were described. The year 2006 was included to compare the data before and after the introduction of a postgraduate mandatory training system. Then, a cohort dataset was established using physician registration numbers. The retention rate every 2 years during 1996-2016 was calculated and analyzed. The number of physicians still working as OBGYNs at the time of the subsequent survey (e.g., in 1998) was divided by the number of OBGYNs in the original survey (e.g., in 1996). The square root of biannual rates yielded annual retention rates.

To identify retention-associated factors in 2006 and 2016, a multivariate logistic regression was conducted for respondents identifying themselves as OBGYNs in 1996 and 2006. The dependent variable was continuation as an OBGYN after 10 years. The independent variables were gender, years of experience, qualified as a physician over 30 years of age (i.e., >5 years’ experience, predicted by graduation at age 25 after entering medical college directly from high school), and workplace (geographic area).

In addition, I analyzed the institutions and specialties of physicians who changed careers between 1996 and 2006, as well as between 2006 and 2016. Finally, the specialty certificates held by physicians as of 2016 were verified.

Significance was set at *P* < 0.05. All analyses were conducted using STATA 15.1 (STATA Corp).

This study was approved by the institutional review board of the Harvard T.H. Chan School of Public Health (No. 18-1422). The need for informed consent was waived due to the national mandatory survey.

## Results

Physicians/population was calculated as the number of physicians per 100 000 residents. In 1996, 2006, and 2016, 12 402 (5.2% of all physicians), 11 783 (4.2%), and 13 154 (4.1%) OBGYNs were practicing in Japan, respectively (Table 1). During this period, the number of physicians increased by 6%. Moreover, the number of urban OBGYNs increased by 15%, whereas that of rural OBGYNs decreased by 25%; thus, the gap between urban and rural practitioners increased.

**Table 1.** Demographic and Professional Characteristics of OBGYNs in 1996, 2006, and 2016.

	1996 Survey		2006 Survey		2016 Survey	
**Total Subjects, n**	12 402		11 783		13 154	
**% of all physicians**	5.2		4.2		4.1	
**Sex, n, %**						
**Male**	10 481	84.5%	9015	76.5%	8473	64.4%
**Female**	1921	15.5%	2768	23.5%	4681	35.6%
**Age, n, %**						
**≦39**	3812	30.7%	3201	27.2%	3771	28.7%
**40-54**	3620	29.2%	3968	33.7%	4170	31.7%
**55-69**	3299	26.6%	2654	22.5%	3739	28.4%
**≧70**	1671	13.5%	1960	16.6%	1474	11.2%
**Years of experience, n, %**						
**0-14**	4106	33.1%	3336	28.3%	3978	30.2%
**15-29**	3567	28.8%	4046	34.3%	4144	31.5%
**30-44**	2820	22.7%	2611	22.2%	3502	26.6%
**≧45**	1909	15.4%	1790	15.2%	1530	11.6%
**Qualified over 30 years old, n, %**						
**<30**	8847	71.3%	8621	73.2%	9812	74.6%
**≧30**	3555	28.7%	3162	26.8%	3342	25.4%
**Workplace, n, %**						
**Urban**	5855	47.2%	5620	47.7%	6708	51.0%
**Intermediate**	5621	45.3%	5402	45.8%	5756	43.8%
**Rural**	926	7.5%	761	6.5%	690	5.2%
**Institution, n, %**						
**Clinic**	5413	43.6%	5403	45.9%	5342	40.6%
**Academic hospital**	2035	16.4%	1793	15.2%	2328	17.7%
**Other hospital**	4954	39.9%	4587	38.9%	5484	41.7%

Between 1996 and 2016, the number of female physicians increased 2.4-fold, whereas their proportion increased from 15.5% to 35.6% (Table 1). The number of OBGYNs aged ≤39 and 55-69 dropped from 1996 to 2006, but rose from 2006 to 2016, whereas OBGYNs aged 40-54 consistently increased during both periods. Contrarily, the number of OBGYNs aged ≥70 increased from 1996 to 2006 and dropped from 2006 to 2016. The number of practicing OBGYNs with 0-14 years of experience fell from 3860 in 2002 to 3336 in 2006. Finally, while the number of OBGYNs working at clinics remained steady, the number of those working at academic or other hospitals decreased from 1996 to 2006, before recovering from 2006 to 2016.

I found that 91.1% of OBGYNs at one agency in 2014 remained there 2 years later (Table 2). In addition, the retention percentage did not change much during the 1996-2016 period, increasing from 88.1% to 91.1%.

**Table 2.** Retention Rate among Obstetricians and Gynecologists.

	Period and number observed										
	1996-1998	1998-2000	2000-2002	2002-2004	2004-2006	2006-2008	2008-2010	2010-2012	2012-2014	2014-2016	2016
**Number of baseline** **OBGYNs, n (%)**	12 402 (100)	12 457 (100)	12 371 (100)	12 329 (100)	12 156 (100)	11 783 (100)	11 960 (100)	12 368 (100)	12 707 (100)	12 888 (100)	13 154
**Still working as physician** **scientists, n (%)**	10 878 (87.7)	10 818 (86.8)	10 684 (86.4)	10 748 (87.2)	10 560 (86.9)	10 329 (87.7)	10 650 (89)	11 027 (89.2)	11 375 (89.5)	11 618 (90.1)	
**Change in area of practice, n (%)**	654 (5.3)	618 (5)	609 (4.9)	617 (5)	584 (4.8)	502 (4.3)	358 (3)	407 (3.3)	387 (3)	372 (2.9)	
**Entered in area of** **practice, n (%)**	1579 (12.7)	1553 (12.5)	1645 (13.3)	1408 (11.4)	1223 (10.1)	1631 (13.8)	1718 (14.4)	1680 (13.6)	1513 (11.9)	1536 (11.9)	
**No report, n (%)**	870 (7)	1021 (8.2)	1078 (8.7)	964 (7.8)	1012 (8.3)	952 (8.1)	952 (8)	934 (7.6)	945 (7.4)	898 (7)	
**Estimated annual retention rate, %**	89.1	88.5	88.1	88.7	88.5	89.1	90.2	90.3	90.6	91.1	
**Retention rate by number ** **of years since registration ** **as a physician, %**											
**0-14**	90.4	89.3	88.2	89.1	88.6	89.2	89.2	90.4	90.7	91.6	
**15-29**	93.9	93.7	93.5	93.8	93.9	93.7	94.5	94.8	94.8	94.5	
**30-44**	87.1	86.8	87.3	87.9	88.3	90.6	91.4	91.5	91.2	92.6	
**≧45**	81.9	81	80.5	80.5	79.4	79	81.3	79.2	79.9	79.6	

OBGYN, obstetricians and gynecologist

Especially among older physicians with over 45 years of experience, the retention rates were relatively low, unchanged from over 81.9% in 1996 to 79.6% in 2014. The ratios of those who did not report 2 years later (no-report ratio) ranged between 7.0% (2014) and 8.7% (2000), indicating no significant differences between the surveys.

The logistic regression indicated that having 30-44 or over 45 years of experience (reference: 0-14 years) and working in a rural area (reference: urban area) were negatively associated with continuation as an OBGYN in both the 1996-2006 and 2016 cohorts (Table 3). In only the 1996-2006 cohort, the negative predictors were 15-29 years of experience (reference: 0-14 years) and working in an academic hospital or other types of hospitals (reference: clinic).

**Table 3.** Logistic Regression Analysis of Factors Associated with the Retention of Obstetricians and Gynecologists.

	1996-2006 cohort				2006-2016 cohort		
	OR	CI	P-value		OR	CI	P-value
**Sex**				**Sex**			
**Male**	Reference			**Male**	Reference		
**Female**	0.93	0.77-1.12	0.44	**Female**	0.85	0.69-1.05	0.13
**Years of experience**				**Years of experience**			
**0-14**	Reference			**0-14**	Reference		
**15-29**	0.77	0.64-0.93	0.01	**15-29**	1.09	0.85-1.41	0.50
**30-44**	0.20	0.17-0.25	<0.01	**30-44**	0.32	0.25-0.41	<0.01
**≧45**	0.14	0.11-0.17	<0.01	**≧45**	0.11	0.08-0.15	<0.01
**Qualified over 30 years old**				**Qualified over 30 years old**			
**No**	Reference			**No**	Reference		
**Yes**	0.89	0.78-1.02	0.10	**Yes**	0.79	0.66-0.96	0.02
**Workplace**				**Workplace**			
**Urban**	Reference			**Urban**	Reference		
**Intermediate**	0.91	0.79-1.04	0.16	**Intermediate**	0.93	0.79-1.10	0.42
**Rural**	0.65	0.51-0.82	<0.01	**Rural**	0.59	0.43-0.80	<0.01
**Institution**				**Institution**			
**Clinic**	Reference			**Clinic**	Reference		
**Academic hospital**	0.67	0.87-1.31	<0.01	**Academic hospital**	0.85	0.63-1.15	0.30
**Other hospital**	0.66	0.84-1.17	<0.01	**Other hospital**	0.84	0.69-1.01	0.07

In 2006 and 2016, most physicians who stopped being OBGYNs worked in hospitals and clinics (Table 4). Other destinations (transfer to nursing or geriatric care facility, leaving the workforce) constituted over a third of the responses at both time points. In addition, more physicians in the 1996-2006 and 2006-2016 cohorts were working as internists after they stopped being OBGYNs compared with any other discipline, followed by psychiatrists (Table 4).

**Table 4.** Types of Institutions and Specialties for Those Who Left Obstetricians and Gynecologists.

	*n*	%
**1996-2006 cohort**		
**Total**	1216	100
**Clinic**	431	35.4
**Academic hospital**	23	1.9
**Other hospitals**	327	26.9
**Others**	435	35.8
**2006-2016 cohort**		
**Total**	691	100
**Clinic**	231	33.4
**Academic hospital**	19	2.7
**Other hospital**	197	28.5
**Others**	244	35.3
	***n***	**%**
**1996-2006 cohort**		
**Total**	9792	100
**OBGYN**	8576	87.6
**Internal medicine**	480	4.9
**Psychiatry**	34	0.3
**Ophthalmology**	29	0.3
**Pediatrics**	23	0.2
**Others**	650	6.6
**2006-2016 cohort**		
**Total**	9376	100
**OBGYN**	8685	92.6
**Internal medicine**	202	2.2
**Psychiatry**	25	0.3
**General surgery**	12	0.1
**Pediatrics**	11	0.1
**Others**	441	4.7

OBGYN, obstetricians and gynecologist.

A total of 2314 physicians (18% of the 13 154 physicians working as OBGYNs) did not hold a specialist qualification. The number of physicians qualified as specialists was the highest among OBGYNs, followed by cytology, gynecologic tumor, perinatal care, and reproductive medicine (Table 5). Data on specialists in 1996 and 2006 are not available because collection of such information did not begin until 2010.

**Table 5.** Board Certification for Obstetricians and Gynecologists in 2016.

	*n*	%
**Total**	13 154	100
**No certificate**	2314	17.6
**OBGYN**	10 733	81.6
**Cytology**	738	5.6
**Gynecologic tumor**	665	5.1
**Perinatal care**	475	3.6
**Reproductive medicine**	449	3.4

OBGYN, obstetricians and gynecologist.

## Discussion

This study revealed that the number of OBGYNs significantly increased between 1996 and 2016. However, rural areas saw a smaller increase than urban areas, which means that geographical maldistribution worsened. Thus, my work confirms the increasing trends previously observed in OBGYNs ^(13)^.

The number of female OBGYNs has consistently increased over time, accounting for 35.6% of the specialty in 2016. Women account for a greater percentage of OBGYNs compared with other specialties; in the United States, 43% of OBGYNs in clinical practice as of 2007 are female, indicating an upward trend ^(18)^. If recent patterns are any indication, the proportion of female OBGYNs in Japan should continue to rise, demanding policies that can support the needs of women. These include flexible working arrangements to account for pregnancy and childcare, as well as comprehensive training upon returning to the workforce after maternity/childcare leave ^(19)^.

Previous research revealed that the number of new doctors specializing as OBGYNs is declining ^(13)^. However, my data here indicate a reversal of this trend from 2006 to 2016, as junior physicians (39 years or younger) joined the workforce. Until 2003, budding physicians chosen to specialize as OBGYNs during the first year after graduating medical school. In 2004, Japan’s medical education system was revised to implement mandatory postgraduate clinical training for 2 years. Therefore, new OBGYNs who started working in 2006 or later could only do so in the third year after matriculation ^(20)^.

The number of practicing OBGYNs with 0-14 years of experience fell from 3860 in 2002 to 3336 in 2006 (Table 1). This drop reflects the legal inability of first-year medical school graduates to specialize as OBGYNs in 2004 and 2005. However, their subsequent rise seems to suggest successful efforts to retain OBGYNs, led by the JSOG and related organizations ^(10)^.

With the exception of a decline among older, senior OBGYNs, the retention rates for the profession as a whole are on the rise. While the ranks of female physicians have swelled, along with the number of doctors who might temporarily put their careers on hold for childbirth or childcare ^(5)^, OBGYNs exhibited a retention rate of over 90% in recent years. These findings suggest that to increase the number of practicing female OBGYNs, efforts should be made to prevent them from leaving entirely while retaining new doctors entering the specialty.

Through logistic regression, I identified the factors that predict continued OBGYN practice for over 10 years. Long-term (≥30 years) careers in medicine and working in a rural area were associated with an increased likelihood of discontinuation. Doctors practicing in rural areas likely shoulder a greater occupational burden than their urban counterparts, which may encourage them to quit.

Physicians who transitioned from OBGYN to another specialty chose internal medicine. This decision may reflect the fact that their responsibilities do not involve surgery and can be handled with comparative ease, even by older physicians ^(21)^. This may be similar in any other surgical specialty. For example, another study reported that neurosurgeons and orthopedic surgeons may quit surgery and become a doctor for rehabilitation ^(22)^.

Most OBGYNs (82%) had some form of board certification in 2016. Statistics from the MHLW indicate that the number of physicians with specialist certifications is on the rise (61% in 2018) ^(12)^. The fact that certifications were more common among OBGYNs than among other physicians indicates that the group is strongly motivated to acquire and maintain their professional qualifications.

In Japan, infant deliveries have historically been decentralized, spread across many small-scale units ^(10)^. However, the reduction and consolidation of such centers have continued unabated in recent years ^(23)^. The factors driving these changes may include accelerated OBGYN migration to urban medical centers, in addition to the societal demands identified in proposals issued by the JSOG and JAOG ^(11)^.

I should also mention that OBGYNs have some of the longest working hours among physicians ^(24), (25)^ and low career satisfaction ^(25)^. Thus, continuous and stable OBGYN retention requires improved working environments, for example, through task shifting and further centralization of OBGYN institutions ^(3), (24)^.

Researchers have pointed to the shorter working hours of female physicians compared with their male counterparts as one potential factor contributing to the shortage of OBGYNs ^(5)^. My study cannot comment on this point, as the OBGYN data utilized in this study did not include comprehensive information on subspecialty, working hours, shift work availability, or engagement in delivery. Thus, my simplistic analysis―how raw numbers of practicing OBGYNs have been distributed over time―may fail to fully capture variables driving the observed shortage. From the 2020 doctor survey, information on whether physicians are working full-time or part-time and whether or not they are engaged in childbirth will be added; detailed data could help elucidate this issue.

In this study, I focused the discussion on OBGYN; however, similar problems may be present in any other specialties. Particularly, the lack of doctors in rural areas has become a problem in Japan. The data in the present study may be utilized as reference for future studies to investigate the distribution trends of other doctors in Japan.

This study has several limitations. First, the area of practice was self-reported; thus, misclassification might have occurred. Second, I did not obtain data for part-time OBGYNs. Third, due to the secondary use of existing data, I could not consider potential variables that might explain physicians’ area-of-practice choices; these include place of origin, graduating university, salary, and family structure ^(26)^. Fourth, this study only determined correlations and not causality. The use of interviews and questionnaires could facilitate more comprehensive research. Fifth, I categorized SMAs into three based on population size and density. Therefore, changing the classification method could alter the results. Despite these limitations, my study also has a major strength in its large-sample cohort.

In conclusion, this study found that from 1996 to 2016 in Japan, the number of practicing OBGYNs increased with the increase in female physicians. Newly implemented mandatory postgraduate clinical training caused a drop in the number of young doctors in 2004, which reversed by 2006. Rural to urban migration was steady, and the working hours were consistently long. The results indicate that the working environments should be improved to stabilize high retention rates.

This study is significant because prior to my work, little was known about OBGYN retention trends and the contributing factors. This study provides useful information for further discussion about OBGYN maldistribution. I recommend that future research should examine physicians’ career-choice rationale, which should identify problem areas that could be targeted to keep physicians on the OBGYN path.

## Article Information

### Conflicts of Interest

None

### Approval by Institutional Review Board (IRB)

18-1422; Harvard T.H. Chan School of Public Health
